# The human C-type lectin 18 is a potential biomarker in patients with chronic hepatitis B virus infection

**DOI:** 10.1186/s12929-018-0460-2

**Published:** 2018-07-28

**Authors:** Tsung-Yu Tsai, Cheng-Yuan Peng, Hwai-I Yang, Ya-Lang Huang, Mi-Hua Tao, Shin-Sheng Yuan, Hsueh-Chou Lai, Shie-Liang Hsieh

**Affiliations:** 1 Ph.D. Program for Translational Medicine, China Medical University and Academia Sinica, Taichung and Taipei, Taiwan; 2 Division of Hepatogastroenterology, Department of Internal Medicine, China Medical University Hospital, 2, Yude St., North District, Taichung, 404 Taiwan; 3 School of Medicine, China Medical University, Taichung, Taiwan; 4 Genomics Research Center, Academia Sinica, 128, Academia Road, Sec. 2, Nankang District, Taipei, 115 Taiwan; 5 Institute of Biomedical Sciences, Academia Sinica, 128, Academia Road, Sec. 2, Nankang District, Taipei, 115 Taiwan; 6 Institute of Statistical Sciences, Academia Sinica, 128, Academia Road, Sec. 2, Nankang District, Taipei, 115 Taiwan; 7 Institute of Clinical Medicine, National Yang-Ming University, Taipei, Taiwan; 8 Department of Medical Research, Taipei Veterans General Hospital, Taipei, Taiwan

**Keywords:** Hepatitis B virus, C-type lectin 18, HBeAg seroconversion

## Abstract

**Background:**

Hepatitis B virus (HBV) infection is a common disease worldwide and is known to cause liver disease. C-type lectin 18 (CLEC18) is a novel secretory lectin highly expressed in human hepatocytes. Because the liver is the major target of HBV infection, we investigated whether the expression of CLEC18 can be used as a biomarker for HBV infection.

**Methods:**

The expression level of CLEC18 in human liver chimeric mice with/without HBV infection was measured by quantitative real time polymerase chain reaction (qPCR) assay. Baseline plasma CLEC18 levels in 271 treatment-naive patients with chronic hepatitis B (CHB) undergoing nucleos(t)ide analogue (NUC) therapy and 35 healthy donors were measured by enzyme-linked immunosorbent assay, and the relationships to other clinical data were analyzed.

**Results:**

The expression of CLEC18 was down-regulated in the human liver chimeric mice after HBV infection. Plasma CLEC18 levels were lower in the patients with CHB compared to the healthy donors and positively correlated with HBV DNA and HBsAg levels (*P* <  0.05). Multivariate Cox proportional hazard regression analysis identified a baseline plasma CLEC18 level of 320–2000 pg/mL to be an independent predictor of HBeAg loss (hazard ratio (HR): 2.077, *P* = 0.0318), seroconversion (HR: 2.041, *P* = 0.0445) and virological response (HR: 1.850, *P* = 0.0184) in 101 HBeAg-positive patients with CHB undergoing NUC therapy.

**Conclusions:**

Plasma CLEC18 levels were correlated with the stage of HBV infection and could predict HBeAg loss and seroconversion in the patients with CHB undergoing NUC therapy.

**Electronic supplementary material:**

The online version of this article (10.1186/s12929-018-0460-2) contains supplementary material, which is available to authorized users.

## Background

Hepatitis B virus (HBV) infection is a global health problem. Current treatment options for hepatitis B e antigen (HBeAg)-positive HBV-infected patients include interferon therapy and nucleos(t)ide analogues (NUCs). HBeAg loss and seroconversion are defined as an intermediate therapeutic endpoint in HBeAg-positive patients [1, 2]. Evaluating the treatment outcome, the status of liver fibrosis is important for patients with chronic hepatitis B (CHB). Clinically, a decline in HBV DNA levels during treatment and high serum alanine aminotransferase (ALT) level can predict HBeAg loss and HBeAg seroconversion [3]. A liver biopsy is the gold standard method to assess the stage of liver fibrosis, although it has the disadvantage of a high complication rate. Noninvasive methods using biomarkers such as hepatitis B surface antigen (HBsAg), serum ALT levels [4], and scoring systems such as fibrosis-4 (FIB-4) and aspartate aminotransferase (AST) to platelet ratio index (APRI) have also been proposed [5, 6]. However, these methods have limitations as independent disease markers [7, 8]. In addition, other immune markers such as tumor necrosis factor-alpha (TNF-α), programmed cell death protein-1 (PD-1) [9] and serum markers such as apolipoprotein and haptoglobulin are not specific for HBV disease and can easily be influenced by other diseases [8]. Biomarkers to assess the treatment outcome of HBV infection and liver fibrosis are still under development.

C-type lectin 18 (CLEC18) is a novel secretory C-type lectin, and we previously showed that CLEC18 is highly expressed in the liver. It is localized in the endoplasmic reticulum, Golgi apparatus, and endosomes, and it can be detected in human plasma by enzyme-linked immunosorbent assay (ELISA). CLEC18 is secreted into the culture supernatant of innate immune cells such as monocytes, dendritic cells and macrophages, which suggests that it is related to the function of the innate immune system [10].

HBV can weaken the host immune response without inducing a pattern recognition receptor (PRR)-mediated cytokine response [11, 12]. The mechanisms by which HBV attenuates Toll-like receptor (TLR)-mediated cytokine responses have been investigated [13], however an association between CLEC18 and HBV infection has yet to be elucidated.

The liver is the major target of HBV infection, and CLEC18 is highly expressed in the liver. Therefore, we hypothesized that the expression of CLEC18 would be influenced by HBV infection. The aim of this study was to investigate the expression of CLEC18 in the liver and its potential role as a biomarker for HBV infection.

## Methods

### Infection of human liver chimeric mice with HBV

Human liver chimeric mice were generated from Fah−/−/Rag2−/−/Il2rg−/− mice (FRG mice) with transplanted human hepatocytes (kindly provided by Dr. Mi-Hua Tao) [14–16]. Each human liver chimeric mouse was infected with HBV, produced by HBV transgenic mice using the hydrodynamic vein injection method as described previously [14]. In brief, 6-week-old FRG mice were intrasplenically transplanted with human hepatocytes (BD Biosciences, USA). HBV obtained from ICR/HBV transgenic mice was hydrodynamically injected into the FRG mice after 3–4 months of transplantation, as previously described [17]. The mice were then sacrificed at 10 and 26 weeks after HBV infection, and liver samples were collected for analysis.

### CLEC18 detection in the human liver chimeric mice with/without HBV infection

Total ribonucleic acid (RNA) was extracted from liver tissues using Trizol according to the manufacturer’s instructions (Invitrogen, USA). The RNA was subjected to reverse transcription using a RevertAid™ First Strand complementary DNA (cDNA) Synthesis Kit (Fermentas), and was then used as the template for polymerase chain reaction (PCR) amplification. CLEC18 cDNA levels in the liver tissue were quantified by real-time PCR using hybridization probes (Roche Life Science, CH) with a thermocycler (LightCycler480®II, Roche, CH) as previously described [13].

### Patients

We enrolled 271 treatment-naïve patients with CHB (101 positive and 170 negative for HBeAg) who received NUC treatment with indications according to the guidelines of the Asian Pacific Association for the Study of the Liver (APASL) at the Hepatology Clinic of China Medical University Hospital in Taichung, Taiwan from August 2005 to August 2016 [2]. The inclusion criteria were age ≥ 20 years and a history of HBsAg carriage for more than 6 months. The exclusion criteria were coinfection caused by other etiologies such as hepatitis C virus, hepatitis D virus, or human immunodeficiency virus; decompensated liver disease; other forms of liver disease; hepatocellular carcinoma at baseline; coexisting severe medical diseases or cancer; and the concurrent use of immunomodulatory drugs or corticosteroids. Among the 101 HBeAg-positive patients, 80 received entecavir, 17 received tenofovir, three received telbivudine, and one received lamivudine. Of these patients, 56 achieved HBeAg loss and 36 achieved HBeAg seroconversion. All of these patients received NUC therapy until the end of the follow-up period of this study, and none experienced viral resistance. Plasma was stored in − 80 °C refrigerators. We also enrolled 35 healthy donors who volunteered for blood donation (17 men and 18 women, 14 aged > 40 years and 21 aged < 40 years). Healthy donors were defined as those who did not have any chronic diseases or cancer and had normal annual health examination reports, including ALT.

### Laboratory examinations

Baseline plasma CLEC18 levels were measured retrospectively using our inhouse ELISA (being licensed to Biolegend) and ELISA from CUSABIO Life Science. We tested both ELISA kits with recombinant proteins and healthy donor sera. Both ELISA kits had a lower limit of detection of 0.078 ng/mL and correlated with each other well. Platelets, prothrombin time (PT), and serum levels of albumin, total bilirubin, creatinine, alpha-fetoprotein (AFP), ALT and serum HBV DNA were measured at baseline. HBeAg and anti-HBe antibodies (Architect i2000 assay; Abbott Diagnostics, Abbott Park, IL, USA) were detected before treatment in order to categorize the patients as being HBeAg positive or negative. HBeAg and anti-HBe antibodies were detected at baseline and every 3 months during treatment in the HBeAg-positive group. HBsAg levels were quantified retrospectively in the patients enrolled before September 2009 and prospectively in those enrolled thereafter using Abbott Architect HBsAg QT assays (dynamic range, 0.05–250 IU/mL) at baseline and annually thereafter. Serum HBV DNA levels were measured at baseline, 3, 6, and 12 months; and then every 6 months thereafter.. HBV genotyping was performed as previously described [18]. Liver fibrosis (F) was staged according to the METAVIR system [19]. Cirrhosis was defined by one of the following: 1) presence of cirrhosis-related complications such as ascites and esophageal or gastric varices; 2) ultrasonographic evidence of a nodular surface and coarse echotexture of the liver with ascites and/or splenomegaly; and 3) histology. Fatty liver was defined on the basis of repeated ultrasonographic findings with increased echogenicity in the liver. Liver biopsies in these patients were performed before NUC therapy.

### Definition of cutoff values

We stratified the patients into three subgroups according to the values close to the cutoff values of baseline plasma CLEC18 (319.52 and 2015.08 pg/mL, risk estimate: 0.297) and HBsAg levels (2889.3–12,022.2 IU/mL, risk estimate: 0.366) which were associated with the highest rates of HBeAg loss in the patients with CHB receiving NUC therapy using classification and regression tree (CART) analysis (see Additional File 1). We defined the cutoff for age (40 years) and HBV DNA (8.3 log_10_ IU/mL) as the median of the 101 patients, and the cutoff for ALT (5× upper limit of normal (ULN)) according to a previous study [20]. The cutoff values for total bilirubin, PT, platelet, and AFP were based on normal values, and those for APRI and FIB-4 were based on previous reports [8, 9, 21].

### Therapeutic endpoints

HBeAg loss was defined as the absence of serum HBeAg during NUC treatment, and HBeAg seroconversion was defined as HBeAg loss with the presence of anti-HBe antibodies. Virological response was defined as undetectable serum HBV DNA.

### Statistical analysis

Continuous variables were compared between two groups using the Student’s t-test (T), labeled as “T” in Table 1, and presented as the mean ± standard deviation (SD). Categorical variables were analyzed using the chi-squared test, labeled as “C” in Table 1. Linear regression analysis was used to identify factors associated with CLEC18 expression. Cox proportional hazard regression analysis was used to identify factors associated with HBeAg loss, seroconversion, and virological response. Logistic regression analysis was used to identify factors associated with liver pathological fibrosis stage. Kaplan-Meier analysis and the log-rank test were used to compare the cumulative incidence rates of HBeAg loss and seroconversion in subgroups of patients with CHB. SAS version 9.4 (SAS Institute, Inc., Cary, NC, USA) and SPSS (IBM Corp. Released 2013, IBM SPSS Statistics for Windows, Version 22.0. Armonk, NY, USA) were used for statistical analyses. A two-sided *P* value of < 0.05 was considered to be statistically significant.

**Table 1:** Baseline patient characteristics

Variables	Total	HBeAg-negative	HBeAg-positive	P Value
Mean ± SD or N (%)	(*n* = 271)	(*n* = 170)	(n = 101)	
Age	47.39 ± 11.35	51.57 ± 10.45	40.37 ± 10.44	< 0.0001^T^
Gender				0.6454^C^
Man	187 (69.0)	119 (70.0)	68 (67.33)	
Woman	84 (31.0)	51 (30.0)	33 (32.67)	
Genotype				0.0003^C^
B	166 (62.88)	118 (71.08)	48 (48.98)	
C	98 (37.12)	48 (28.92)	50 (51.02)	
HBsAg: log_10_ IU/mL	3.34 ± 0.84	3.04 ± 0.89	3.85 ± 0.66	< 0.0001^T^
HBV DNA: log_10_ IU/mL	6.89 ± 2.16	5.91 ± 1.84	8.56 ± 0.93	< 0.0001^T^
Cirrhosis				0.0028^C^
No	182 (67.16)	103 (60.59)	79 (78.22)	
Yes	89 (32.84)	67 (39.41)	22 (21.78)	
Fatty liver				0.2510^C^
No	133 (49.08)	88 (51.76)	45 (44.55)	
Yes	138 (50.92)	82 (48.42)	56 (55.45)	
Albumin: g/dL	4.07 ± 0.51	4.04 ± 0.54	4.10 ± 0.48	0.5446^T^
ALT: IU/L	281.5 ± 411.2	210.8 ± 333.2	400.5 ± 472.3	0.0002^T^
Total bilirubin: mg/dL	1.40 ± 1.34	1.32 ± 0.95	1.54 ± 1.54	0.7096^T^
Platelet: × 10^3^/μL	161.0 ± 60.73	149.4 ± 65.8	180.8 ± 58.1	0.0004^T^
PT: seconds prolonged	1.71 ± 2.00	1.69 ± 1.73	1.86 ± 1.91	0.3551^T^
Cr: mg/dL	0.88 ± 0.49	0.90 ± 0.61	0.86 ± 0.40	0.2857^T^
AFP: ng/mL	25.31 ± 75.62	21.24 ± 44.57	32.17 ± 89.88	0.4094^T^
Numbers of liver biopsy	164	105	59	
METAVIR Activity grade				0.5302^C^
0,1	97 (59.15)	64 (60.95)	33 (55.93)	
2,3	67 (40.85)	41 (39.05)	26 (44.07)	
METAVIR Fibrosis stage				0.0949^C^
0–2	91 (57.59)	52 (52.53)	39 (66.10)	
3,4	67 (42.41)	47 (47.47)	20 (33.90)	

*T* Student’s T-test, *C* Chi-squared test

## Results

### Down-regulation of the expression of CLEC18 in the liver

**Fig. 1:**
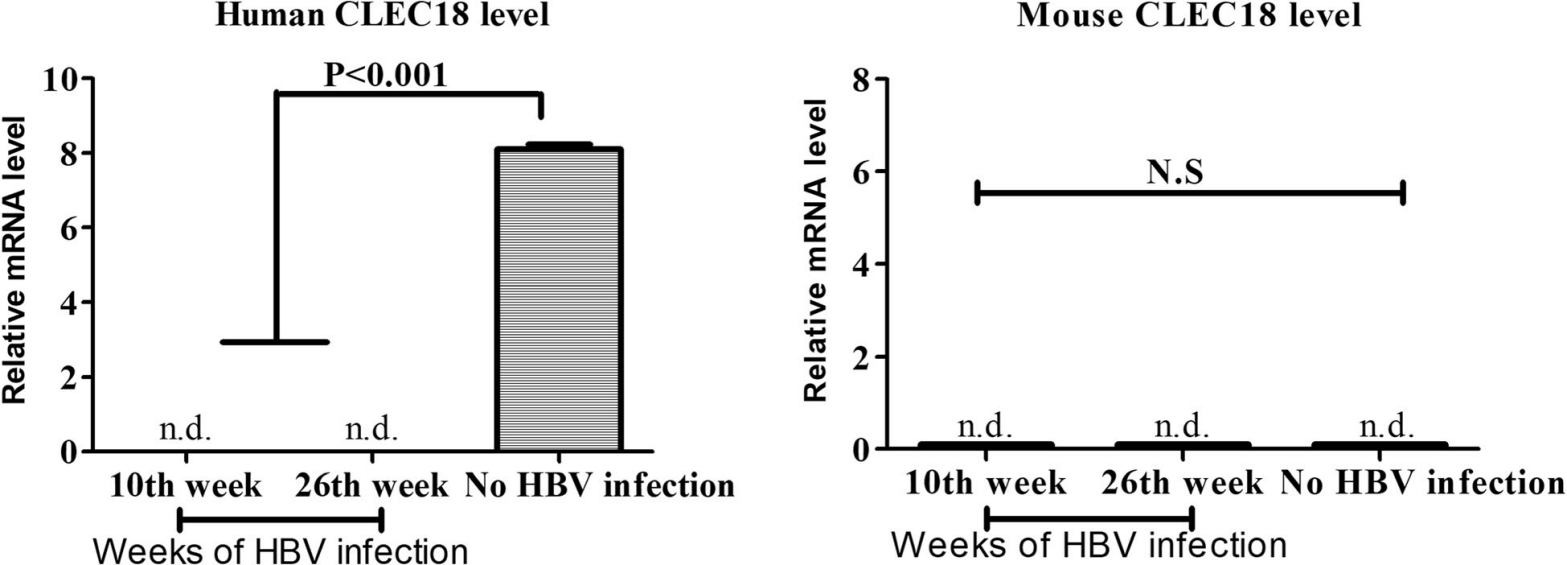
Liver CLEC18 expression in human liver chimeric mice with HBV infection. Total RNA was extracted from liver tissues in liver chimeric mice at 10 and 26 weeks of HBV infection. The RNA was subjected to reverse transcription into cDNA as described in the “Materials and Methods” section. Liver CLEC18 cDNA level was quantified by real-time PCR using hybridization probes. The non-significant (N.S.) and significant (*P* <  0.001; Student’s t-test) results of statistical analysis were obtained by comparing each group to the non-HBV infected group. All data shown are representative of three independent experiments (number of mice in each group = 3). n.d.: non-detectable

The relative mRNA expression levels of human CLEC18 were dramatically down-regulated in the liver tissues of HBV-infected human liver chimeric mice at 10 and 26 weeks after infection compared to the non-infected controls. Mouse CLEC18 was not detected, indicating that the liver tissue collected was only human (Fig. 1). This finding suggested that HBV down-regulated the expression of CLEC18 in human hepatocytes.

### Baseline patient characteristics

The baseline patient characteristics are presented in Table 1. In brief, the HBeAg-positive patients were significantly younger and had lower prevalence rates of genotype B infection and cirrhosis, a higher platelet count, and higher levels of ALT, HBV DNA, and HBsAg than the HBeAg-negative patients.

### Decreased plasma CLEC18 levels in the patients with CHB

In order to understand the role of CLEC18 in different stages of CHB, we divided the patients with CHB into four groups according to the presence of HBeAg and HBV DNA levels. The mean plasma CLEC18 levels were 3106, 663, 281, 264, and 113 pg/mL in the healthy donors (*n* = 35), treatment-naïve HBeAg-positive CHB patients with HBV DNA >  2.0 × 10^7^ IU/mL (*n* = 101), HBeAg-negative CHB patients with HBV DNA >  2.0 × 10^7^ IU/mL (*n* = 65), DNA 2000–2.0 × 10^7^ IU/mL (*n* = 64), and DNA <  2000 IU/mL (*n* = 41), respectively. The plasma CLEC18 level was significantly lower in each HBV-infected group compared to the healthy donors and in the HBeAg-negative group compared to HBeAg-positive group (*P* <  0.05–0.001) (Table 2). There were no significant changes in plasma CLEC18 levels with different viral loads in the HBeAg-negative patients.

**Table 2:** Plasma CLEC18 levels in the patients with CHB

Group (viral load IU/mL)	Number of patients	CLEC18 level(Mean ± SD pg/mL)	*P* Value^(1)^	*P* Value^(2)^
Healthy donors	35	3106.06 ± 4708.13	–	–
HBeAg-positive (> 2 × 10^7^)	101	663.59 ± 1375.62	< 0.001	–
HBeAg-negative (> 2 × 10^7^)	65	281.68 ± 753.07	< 0.001	0.0196
HBeAg-negative (2000-2 × 10^7^)	64	264.68 ± 553.70	< 0.001	0.0106
HBeAg-negative (< 2000)	41	113. 28 ± 231.69	< 0.001	0.0057

*P* value^(1)^: Compared to healthy donors (Student’s t-test)

*P* value^(2)^:Compared to HBeAg-positive(> 2 × 10^7^) group (Student’s t-test)

### Factors associated with plasma CLEC18 levels in the patients with CHB

We used univariate and multivariate linear regression analyses to identify factors associated with plasma CLEC18 levels in the patients with CHB (Table 3). Univariate analysis revealed that age was negatively associated with plasma CLEC18 levels, and that HBeAg positivity, HBsAg, HBV DNA, and ALT levels were positively associated with plasma CLEC18 levels. Multivariate analysis identified age to be a marginal independent factor associated with plasma CLEC18 levels.

**Table 3:** Factors associated with CLEC18 levels in the patients with CHB

Variables	Univariate analysis				Multivariate analysis			
	Parameter Estimate	Standard Error	T Value	*P* Value	Parameter Estimate	Standard Error	T Value	P Value
Age	−18.41386	5.17495	−3.56	0.0004	−11.3821	6.1275	−1.86	0.0644
Sex: Man vs Woman	−25.76591	128.98391	−0.20	0.8418				
Genotype: C vs B	29.89732	126.53368	0.24	0.8134				
HBeAg: (+) vs (−)	428.94574	120.57574	3.56	0.0004	194.63847	162.600	1.20	0.2324
HBsAg log_10_ IU/mL	189.40927	67.77183	2.79	0.0056	20.737	91.28661	0.23	0.8205
HBV DNA log_10_ IU/mL	85.07892	27.25278	3.12	0.0020	25.6009	40.78698	0.63	0.5308
Cirrhosis: Yes vs No	− 142.57582	126.72962	−1.13	0.2616				
Fatty liver: Yes vs No	−38.09601	119.31101	−0.32	0.7497				
ALT: IU/L	0.30495	0.14451	2.11	0.0358	0.09404	0.16072	0.59	0.5590
Total bilirubin: mg/dL	−5.61291	45.41720	−0.12	0.9017				
Platelet: × 10^3^ /μL	1.16809	0.97747	1.20	0.2331				
PT: seconds prolonged	−19.80886	31.83170	−0.62	0.5343				
Cr: mg/dL	17.03954	124.23906	0.14	0.8910				
AFP: ng/mL	0.99144	0.79197	1.25	0.2117				
METAVIR Activity grade 2, 3 vs 0, 1	− 102.09187	167.92687	−0.61	0.5441				
METAVIR Fibrosis stage 3, 4 vs 0–2	− 303.34073	171.59770	−1.77	0.0791				

### Role of plasma CLEC18 in the prediction of HBeAg loss and seroconversion in the patients with CHB receiving NUC treatment

The overall NUC treatment duration was 59.51 ± 3.21 months for the HBeAg-positive patients. The times to HBeAg loss and seroconversion were 37.69 ± 2.86 months and 45.61 ± 3.26 months, respectively. Among the 101 HBeAg-positive patients, 56 (55.44%) patients experienced HBeAg loss and 36 (35.64%) patients experienced HBeAg seroconversion during NUC treatment.

Univariate analysis identified that baseline ALT level > 5× ULN, AFP >  20 ng/mL, HBsAg level of 2900–12,000 IU/mL, and plasma CLEC18 level of 320–2000 pg/mL were significantly associated with HBeAg loss (Table 4), and that baseline ALT level > 5× ULN, AFP >  20 ng/mL, and plasma CLEC18 level of 320–2000 pg/mL were significantly associated with HBeAg seroconversion (Table 5). Multivariate analysis identified that a baseline plasma CLEC18 level of 320–2000 pg/mL was an independent predictor of HBeAg loss (hazard ratio [HR]: 2.077, 95% confidence interval [CI]:1.066–4.046, *P* = 0.0318) and seroconversion (HR: 2.041, 95% CI: 1.018–4.092, *P* = 0.0445) in the patients with CHB receiving NUC therapy. Baseline HBsAg level could significantly predict HBeAg loss (Table 4).

**Table 4:** Factors associated with HBeAg loss in HBeAg-positive patients

Variables	Univariate analysis		Multivariate analysis	
	Hazard Ratio (95% CI)	P Value	Hazard Ratio (95% CI)	P Value
Age: ≥ 40 vs < 40 years old	0.693 (0.408–1.179)	0.1761		
Sex: Man vs Woman	1.103 (0.609–1.996)	0.7466		
Genotype: C vs B	1.118 (0.661–1.892)	0.6774		
Cirrhosis: Yes vs No	1.150 (0.617–2.144)	0.6591		
HBsAg: 2900–12,000 vs < 2900 or > 12,000 IU/mL	2.696 (1.555–4.673)	0.0004	2.108 (1.133–3.924)	0.0186
HBV DNA: ≥ 8.3 vs < 8.3 log_10_ IU/mL	0.938 (0.534–1.646)	0.8223		
ALT: ≥ 5 × vs < 5 × ULN	2.452 (1.433–4.195)	0.0011	2.055 (1.145–3.690)	0.0158
Total bilirubin: ≥ 1.2 vs < 1.2 mg/dL	1.622 (0.959–2.744)	0.0713		
PT: seconds prolonged	1.130 (0.995–1.282)	0.0592	1.140 (0.981–1.325)	0.0865
Platelet: ≥ 150 vs < 150 × 10^3^/μL	1.551 (0.887–2.710)	0.1234		
AFP: ≥ 20 vs < 20 ng/mL	3.178 (1.732–5.829)	0.0002	2.583 (1.258–5.303)	0.0097
CLEC18: pg/mL	1.000 (0.999–1.000)	0.2708		
CLEC18: 320–2000 vs < 320 or > 2000 pg/mL	2.842 (1.637–4.933)	0.0002	2.077 (1.066–4.046)	0.0318

**Table 5:** Factors associated with HBeAg seroconversion in the HBeAg-positive patients

Variables	Univariate analysis		Multivariate analysis	
	Hazard Ratio (95% CI)	P Value	Hazard Ratio (95% CI)	P Value
Age: ≥ 40 vs < 40 years old	0.700 (0.363–1.351)	0.2878		
Sex: Man vs Woman	0.741 (0.370–1.485)	0.3984		
Genotype: C vs B	1.480 (0.756–2.898)	0.2525		
Cirrhosis: Yes vs No	1.133 (0.532–2.411)	0.7464		
HBsAg: 2900–12,000 vs < 2900 or > 12,000 IU/mL	1.754 (0.897–3.340)	0.1008		
HBV DNA: ≥ 8.3 vs < 8.3 log_10_ IU /mL	0.907 (0.453–1.815)	0.7832		
ALT: ≥ 5 × vs < 5 × ULN	3.115 (1.551–6.254)	0.0014	2.562 (1.241–5.288)	0.0110
Total bilirubin: ≥ 1.2 vs < 1.2 mg/dL	1.392 (0.723–2.682)	0.3228		
PT: seconds prolonged	1.114 (0.955–1.298)	0.1688		
Platelet: ≥ 150 vs < 150 × 10^3^/μL	1.149 (0.565–2.336)	0.7021		
AFP: ≥ 20 vs < 20 ng/mL	2.388 (1.166–4.888)	0.0173	1.950 (0.927–4.103)	0.0783
CLEC18: pg/mL	1.000 (0.999–1.000)	0.3755		
CLEC18: 320–2000 vs < 320 or > 2000 pg/mL	2.609 (1.342–5.072)	0.0047	2.041 (1.018–4.092)	0.0445

**Fig. 2:**
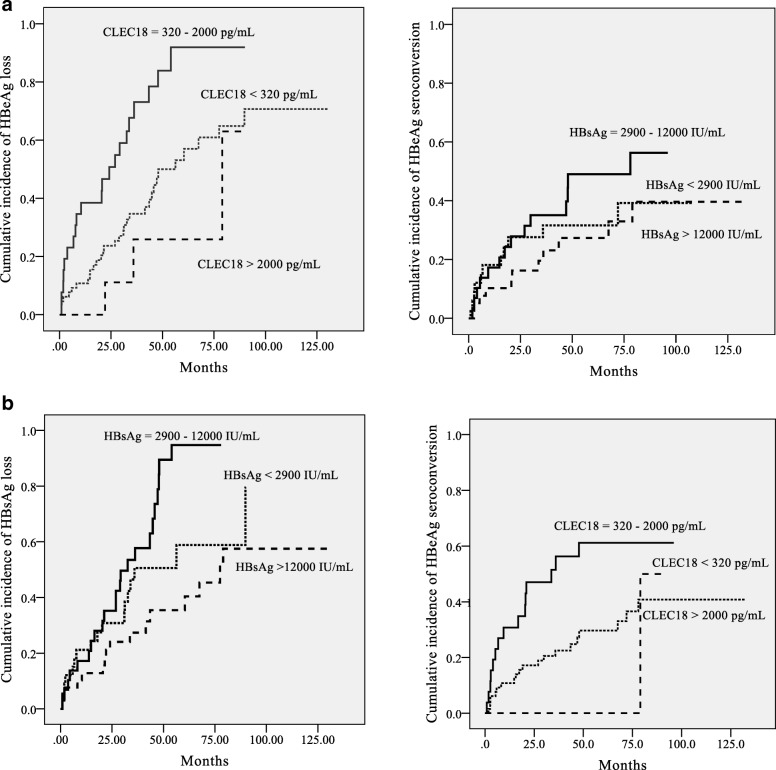
Cumulative incidence of HBeAg loss and seroconversion in the patients with CHB by (**a**) CLEC18 and (**b**) HBsAg levels. Curves of cumulative rates of HBeAg loss and HBeAg seroconversion derived from Kaplan-Meier analysis stratified by (**a**) baseline plasma CLEC18 levels and (**b**) baseline HBsAg levels. Differences between cumulative incidence curves were tested using the log-rank test

The cumulative incidence rates of HBeAg loss and seroconversion in the patients with CHB undergoing NUC therapy with a baseline plasma CLEC18 level of 320–2000 pg/mL were significantly higher than those in the other patients (*P* <  0.001 and *P* = 0.002, respectively) (Fig. 2a). The cumulative incidence of HBeAg loss but not HBeAg seroconversion in the patients with CHB undergoing NUC therapy with a baseline HBsAg level of 2900–12,000 IU/mL was significantly higher than that in the other patients (*P* = 0.029 and *P* = 0.338, respectively) (Fig. 2b).

### Role of plasma CLEC18 in the prediction of virological response in the patients with CHB receiving NUC treatment

The overall NUC treatment duration was 61.68 ± 2.30 months for the HBeAg-negative patients. The times to virological response were 11.48 ± 0.86 months in the HBeAg-positive and 5.56 ± 0.37 months in the HBeAg-negative patients with CHB receiving NUC therapy. In the HBeAg-positive patients, univariate analysis identified that a baseline HBsAg level of 2900–12,000 IU/mL, HBV DNA level <  8.3 log_10_ IU/mL, ALT level > 5× ULN, and plasma CLEC18 level of 320–2000 pg/mL were significantly associated with virological response. Multivariate analysis identified that a baseline plasma CLEC18 level of 320–2000 pg/mL was an independent predictor of virological response (HR: 1.850, 95% CI: 1.109–3.085, *P* = 0.0184) (Table 6). In the HBeAg-negative patients, no factor was significantly associated with virological response.

**Table 6:** Factors associated with virological response in the HBeAg-positive patients

Variables	Univariate analysis		Multivariate analysis	
	Hazard Ratio (95% CI)	P Value	Hazard Ratio (95% CI)	P Value
Age: ≥ 40 vs < 40 years old	0.813 (0.544–1.215)	0.3125		
Sex: Man vs Woman	0.755 (0.488–1.166)	0.2045		
Genotype: C vs B	1.063 (0.707–1.598)	0.7691		
Cirrhosis: Yes vs No	1.257 (0.778–2.031)	0.3502		
HBsAg: 2900–12,000 vs < 2900 or > 12,000 IU/mL	1.698 (1.065–2.706)	0.0260	1.449 (0.890–2.359)	0.1359
HBV DNA: ≥ 8.3 vs < 8.3 log_10_ IU/mL	0.583 (0.378–0.899)	0.0147	0.395 (0.247–0.631)	0.0001
ALT: ≥ 5 × vs < 5 × ULN	1.909 (1.240–2.939)	0.0033	2.191 (1.373–3.496)	0.0010
Total bilirubin: ≥ 1.2 vs < 1.2 mg/dL	1.301 (0.861–1.967)	0.2115		
PT: seconds prolonged	1.015 (0.914–1.128)	0.7751		
Platelet: ≥ 150 vs < 150 × 10^3^/μL	1.297 (0.831–2.025)	0.2525		
AFP: ≥ 20 vs < 20 ng/mL	1.230 (0.742–2.038)	0.4227		
CLEC18: pg/mL	1.000 (1.000–1.000)	0.9850		
CLEC18: 320–2000 vs < 320 or > 2000 pg/mL	1.979 (1.238–3.163)	0.0044	1.850 (1.109–3.085)	0.0184

### Correlation between plasma CLEC18 and baseline HBsAg levels

We analyzed the relationship between CLEC18 and HBsAg levels in the HBeAg-positive patients. CLEC18 and HBsAg levels had a low Spearman’s correlation coefficient (data not shown). The correlation between categorized plasma CLEC18 levels (< 320, 320–2000 and >  2000 pg/mL) and categorized HBsAg levels (< 2900, 2900–12,000 and > 12,000 IU/mL) was not significant (see Additional File 2, *P* = 0.2558).

### The association between plasma CLEC18 levels and liver fibrosis

Of the 271 enrolled patients with CHB, 172 received a liver biopsy. Univariate analysis identified that an age > 40 years, female sex, HBV genotype C, baseline HBsAg < 3.0 log_10_ IU/mL, HBV DNA < 6 log_10_ IU/mL, ALT < 5× ULN, platelet < 150 ×  10^3^/uL were significantly associated with METAVIR fibrosis stages 3 and 4, while CLEC18 showed borderline significance (*P* = 0.0501). Multivariate analysis revealed that a baseline plasma CLEC18 level of < 320 pg/mL was not significantly associated with METAVIR fibrosis stages 3 and 4 (see Additional File 3).

## Discussion

This is the first study to investigate the expression, association and predictive value of CLEC18 in HBV infection. Because it is difficult to obtain liver tissue in patients with CHB, we analyzed the plasma levels of CLEC18 as an alternative.

Plasma CLEC18 decreases during HBV infection. The natural history of chronic HBV infection includes four distinct phases: an immune-tolerant phase, an immune clearance phase, an inactive or residual phase, and a reactivation phase [19]. In the immune-tolerant phase, the patients tend to be younger and have higher HBV DNA levels. As the patients become older, the disease progresses to the inactive phase, and the patients experience HBeAg loss and seroconversion with a decrease in HBV DNA replicative activity [22, 23]. We divided treatment-naïve patients with CHB undergoing NUC therapy into groups to mimic the disease progression of HBV infection. Interestingly, plasma CLEC18 levels were dramatically down-regulated in the patients with CHB (Table 2), suggesting that plasma CLEC18 levels are related to disease progression in HBV infection.

The baseline plasma CLEC18 levels were higher in the HBeAg-positive CHB patients than those in the HBeAg-negative CHB patients. Most of the HBeAg-negative CHB patients had low or even undetectable plasma CLEC18 levels, which precluded further evaluations of the associations with clinical features. The reason why the HBeAg-negative CHB patients exhibited low plasma CLEC18 levels is unknown, however it is possible that HBV infection down-regulates the expression of CLEC18 in the liver, and that long-term chronic infection with HBV results in lower plasma CLEC18 levels.

Previous studies have shown that HBV can attenuate the host immune response [10–12] such as by inhibiting TLR3-mediated cytokine response, which is correlated with the suppression of IFN-β and suppressed activation of IRF-3 and NF-kB [24]. We speculate that CLEC18 may play a role in regulating PRR-mediated cytokine secretion or activating interferon-stimulating genes during viral infection. HBV blocks the expression of CLEC18, which may then result in attenuation of the host immune response. There were no significant correlations between baseline plasma CLEC18 and HBsAg levels, either in linear regression analysis or categorized correlation analysis. However, both may play a role in predicting HBeAg loss and/or seroconversion. The reason why we did not see a significant correlation between baseline plasma HBsAg and CLEC18 levels may be because most of the patients had low plasma CLEC18 levels, which precluded further statistical correlations with HBsAg levels in our study population. Further studies are needed to investigate whether plasma CLEC18 levels are correlated with HBsAg kinetics during NUC treatment. The mechanism by which HBV regulates the expression of CLEC18 during the disease course also remains to be elucidated.

Wang et al. reported that a high baseline HBsAg level (> 10,000 IU/mL) was associated with a lower rate of virological response, and that an on-treatment decline in HBsAg alone was not a good predictor of HBeAg loss and seroconversion in patients with CHB undergoing entecavir treatment [25]. Similar studies have reported that baseline HBsAg level alone or in combination with on-treatment declines in HBsAg, HBeAg, and HBV DNA levels can increase the predictive accuracy of HBeAg seroconversion [26]. However, none of these studies identified a clear cutoff value of HBsAg to predict treatment outcomes in patients receiving NUC treatment. Interestingly, we demonstrated that the subgroups of patients with a baseline HBsAg level of 2900–12,000 IU/mL had a higher likelihood of HBeAg loss and seroconversion. It is possible that the patients with CHB who had already achieved a low HBsAg level of < 2900 IU/mL and remained positive for HBeAg were less likely to lose HBeAg despite treatment, although the underlying immunological and virologic mechanisms remain to be determined. Patients with CHB with a high HBsAg level may have an impaired immune response to HBV owing to the inhibitory effect of viral antigens. The reason why the patients with a baseline HBsAg level of 2900–12,000 IU/mL and CLEC18 level of 320–2000 pg/mL tended to achieve HBeAg loss remains unknown. We speculate that HBV regulates CLEC18 via an unknown pathway in HBeAg-positive CHB patients receiving NUC therapy. This interaction between HBV and CLEC18 may then result in the apparent concordance in the associations between HBsAg and CLEC18 levels and HBeAg loss during NUC therapy. Further investigations into the underlying mechanism are needed. Although a baseline HBsAg level of 2900–12,000 IU/mL was significantly associated with HBeAg loss, it was not significantly associated with a virological response in the HBeAg-positive CHB patients receiving NUC therapy. The reason remains to be investigated.

Taken together, we propose that a range of baseline plasma HBsAg and CLEC18 levels is better than a single cutoff value to predict HBeAg loss and/or seroconversion in NUC-treated HBeAg-positive patients, and that a range of baseline plasma CLEC18 levels can predict a virological response in these patients.

Developing an accurate biomarker to allow for the early management of liver fibrosis is important. In the current study, a plasma CLEC18 level <  320 pg/mL was not significantly associated with liver fibrosis. Whether the level of CLEC18 in the liver reflects liver fibrosis is unknown. Although it was not possible to define liver fibrosis using CLEC18 as a single biomarker, whether CLEC18 can be used in combination with other biomarkers to predict liver fibrosis remains to be elucidated. Further studies are also needed to elucidate whether CLEC18 can be used as a biomarker for liver fibrosis.

There were two limitations to the present study. First, we focused on the prediction of treatment outcomes during NUC therapy, and patients who did not meet the criteria for receiving NUC therapy according to the APASL guidelines [2] were not enrolled, such as immune-tolerant patients (high HBV DNA >  2 × 10^7^ IU/mL, normal ALT levels, HBeAg-positive) and inactive HBsAg carriers (HBsAg-positive, anti-HBe-positive with persistent normal serum ALT levels and HBV DNA <  2000 IU/mL). Further studies enrolling such subgroups of patients are warranted. Second, not all of the HBeAg-positive patients received the same treatment regimen. Nonetheless, the majority of the patients received potent NUCs, with 80 and 17 receiving entecavir and tenofovir, respectively, both of which are first-line therapy as recommended by the APASL guidelines [2].

## Conclusion

Plasma CLEC18 levels were decreased in the patients with CHB and could predict HBeAg loss, seroconversion and virological response in the HBeAg-positive patients with CHB undergoing NUC therapy. Further studies are warranted to clarify the role of CLEC18 in CHB.

## Additional files

Additional file 1: Defined cutoffs of HBsAg and CLEC18 levels for HBeAg loss by CART. (TIF 2684 kb)

Additional file 2: Correlation between HBsAg and CLEC18 levels. (TIF 1367 kb)

Additional file 3: Factors associated with fibrosis stages 3 and 4 in the patients with CHB. (DOCX 22 kb)

## Abbreviations

AFP: Alpha-fetoprotein
ALT: Alanine aminotransferase
APASL: Asian Pacific Association for the Study of the Liver
APRI: AST to platelet ratio index
AST: Aspartate aminotransferase
CART: Classification and regression tree
cDNA: Complementary DNA
CHB: Chronic hepatitis B
CLEC18: C-type lectin 18
ELISA: Enzyme-linked immunosorbent assay
FIB-4: Fibrosis-4
FRG: Fah−/−/Rag2−/−/Il2rg−/−
HBeAg: Hepatitis B e antigen
HBsAg: Hepatitis B surface antigen
HBV: Hepatitis B virus infection
HR: Hazard ratio
NUC: Nucleos(t)ide analogue
PD-1: Programmed cell death protein-1
PRR: Pattern recognition receptor
PT: Prothrombin time
qPCR: Quantitative real time polymerase chain reaction
RNA: Ribonucleic acid
SD: Standard deviation
TLR: Toll-like receptor
TNF-α: Tumor necrosis factor-alpha
ULN: Upper limit of normal
