# Supplementary figures and images for: The human C-type lectin 18 is a potential biomarker in patients with chronic hepatitis B virus infection
Source: J Biomed Sci. 2018 Jul 28;25:59. doi: 10.1186/s12929-018-0460-2 (PMC6064175; doi:10.1186/s12929-018-0460-2)

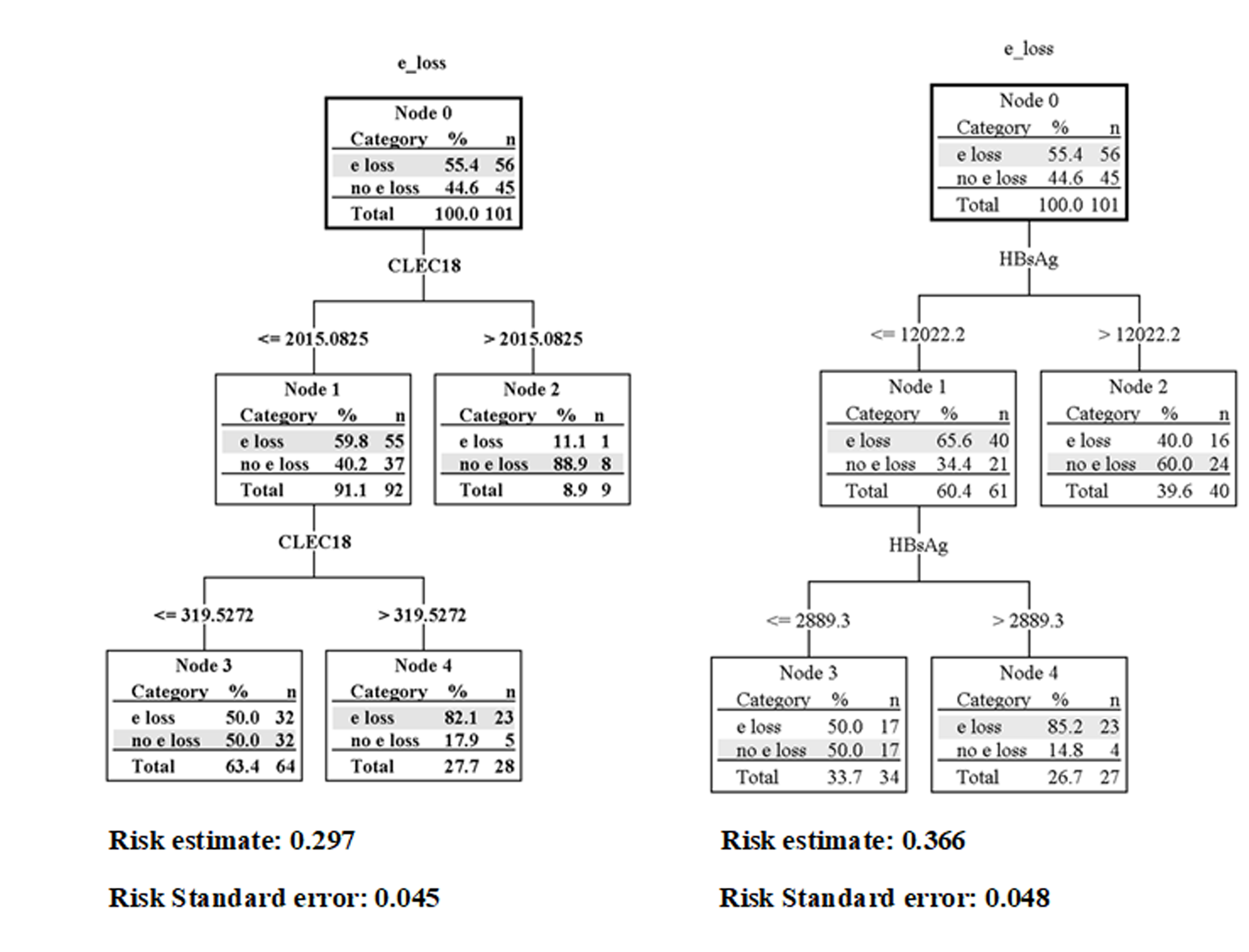

Supplement: Supplementary file 1 — Defined cutoffs of HBsAg and CLEC18 levels for HBeAg loss by CART. (TIF 2684 kb) [file 12929_2018_460_MOESM1_ESM.tif]

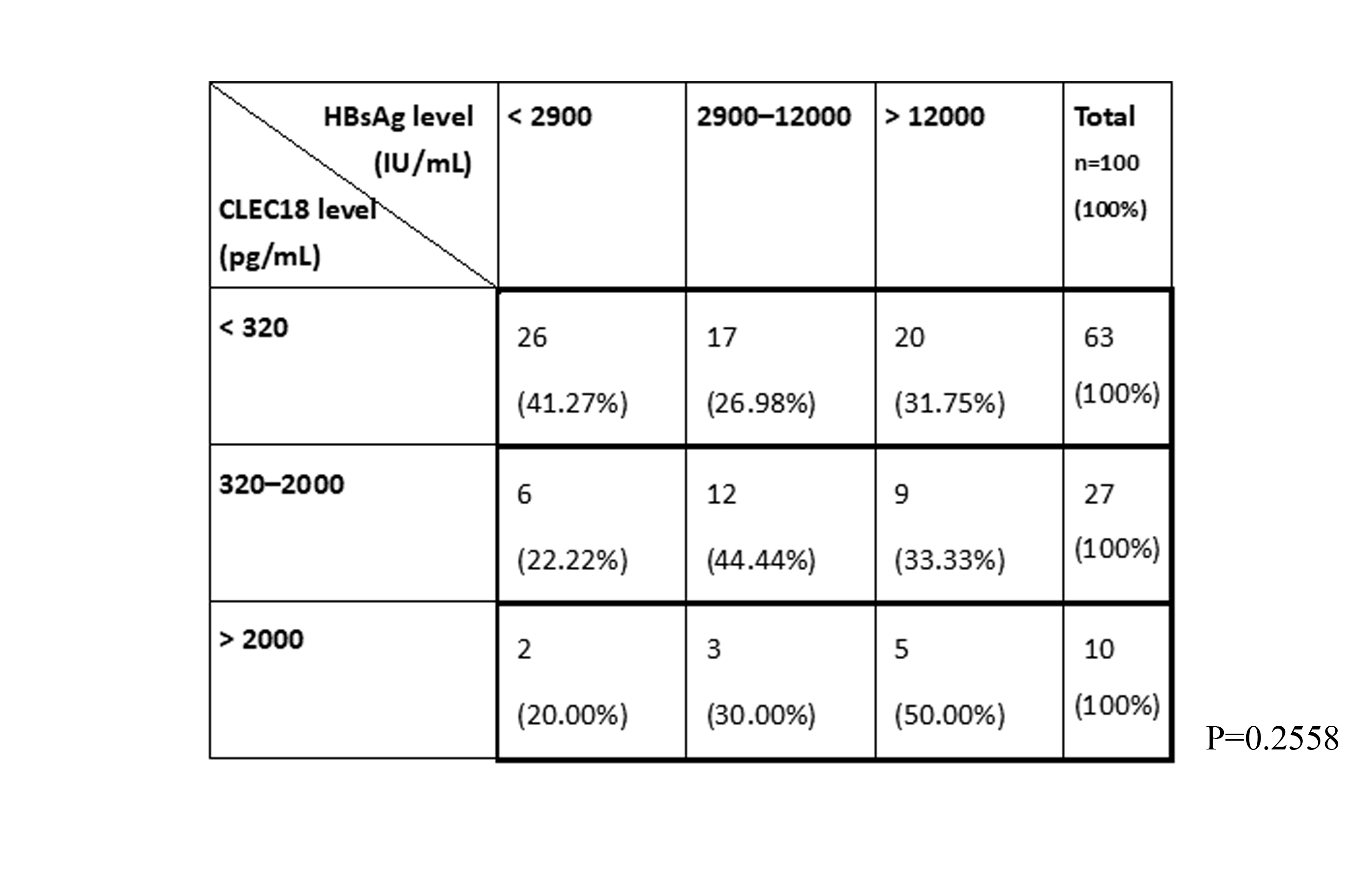

Supplement: Supplementary file 2 — Correlation between HBsAg and CLEC18 levels. (TIF 1367 kb) [file 12929_2018_460_MOESM2_ESM.tif]
