# Supplementary material for: The human C-type lectin 18 is a potential biomarker in patients with chronic hepatitis B virus infection
Source: J Biomed Sci. 2018 Jul 28;25:59. doi: 10.1186/s12929-018-0460-2 (PMC6064175; doi:10.1186/s12929-018-0460-2)
Supplement: Supplementary file 3 — Factors associated with fibrosis stages 3 and 4 in the patients with CHB. (DOCX 22 kb) [file 12929_2018_460_MOESM3_ESM.docx]

**Additional File 3 Factors associated with fibrosis stages 3 and 4 in the patients with CHB**

| **Variables** | **Univariate analysis** | | **Multivariate analysis** | |
| --- | --- | --- | --- | --- |
|  | **Odds Ratio (95% CI)** | **P value** | **Odds Ratio (95% CI)** | **P value** |
| **Age: ≥ 40 vs < 40 years old** | **2.521 (1.126-5.647)** | **0.0246** | **1.079 (0.408-2.858)** | **0.8776** |
| **Sex: Woman vs Man** | **1.266 (0.638-2.512)** | **0.4991** |  |  |
| **Genotype: C vs B** | **1.721 (0.911-3.250)** | **0.0944** |  |  |
| **HBsAg: ≥ 3 vs < 3 log_10_ IU/mL** | **0.218 (0.096-0.492)** | **0.0002** | **0.369 (0.145-0.939)** | **0.0364** |
| **HBV DNA: ≥ 6 vs < 6 log_10_ IU/mL** | **0.334 (0.174-0.641)** | **0.0010** | **0.832 (0.376-1.843)** | **0.6511** |
| **Fatty liver :Yes vs No** | **0.653 (0.351-1.217)** | **0.1799** |  |  |
| **ALT: ≥ 5 × vs < 5 × ULN** | **0.158 (0.068-0.366)** | **0.0001** | **0.206 (0.080-0.532)** | **0.0011** |
| **Total bilirubin: ≥ 1.2 vs < 1.2 mg/dL** | **0.689 (0.356-1.333)** | **0.2687** |  |  |
| **Platelet: ≥ 150 vs < 150 x 10^3^/μL** | **0.254 (0.132-0.490)** | **0.0001** | **0.327 (0.156-0.687)** | **0.0032** |
| **AFP: ≥ 20 vs < 20 ng/mL** | **1.000 (0.997-1.004)** | **0.8145** |  |  |
| **FIB-4: ≥ 1.45 vs < 1.45** | **2.035 (0.945-4.381)** | **0.0694** |  |  |
| **APRI: ≥ 1 vs < 1** | **0.548 (0.295-1.019)** | **0.0573** |  |  |
| **CLEC18: pg/mL** | **1.000 (0.999-1.000)** | **0.0883** |  |  |
| **CLEC18: 320**–**2000 vs**  **< 320 or > 2000 pg/mL** | **0.629 (0.280-1.413)** | **0.2611** |  |  |
| **CLEC18: ≥ 320 vs < 320 pg/mL** | **0.466 (0.217-1.000)** | **0.0501** | **0.519 (0.210-1.285)** | **0.1564** |
